# The effectiveness of intraoperative indocyanine green fluorescence imaging in preventing anastomotic leakage after minimally invasive esophagectomy for esophageal cancer: a systematic review and meta-analysis

**DOI:** 10.3389/fmed.2026.1830155

**Published:** 2026-05-13

**Authors:** Ye-hong Li, Jian-hao Qiu, Zhan Zhang, Tian-run Miao, Yong-meng Li, Hui Tian

**Affiliations:** 1 School of Clinical Medicine, Shandong Second Medical University, Weifang, Shandong, China; 2 Department of Thoracic Surgery, The First Affiliated Hospital of Shandong First Medical University & Shandong Provincial Qianfoshan Hospital, Jinan, China

**Keywords:** anastomotic leakage, esophageal cancer, fluorescence imaging, indocyanine green, minimally invasive esophagectomy

## Abstract

**Background:**

Anastomotic leakage (AL) remains a major complication after minimally invasive esophagectomy (MIE) for esophageal cancer (EC), primarily due to inadequate perfusion at the anastomotic site. Conventional intraoperative assessment of gastric conduit blood supply is subjective and often fails to detect occult hypoperfusion. Although indocyanine green (ICG) fluorescence imaging provides real-time, objective evaluation of Intraoperative tissue perfusion, potentially guiding anastomotic decisions and reducing AL risk, robust evidence supporting its routine use in MIE remains limited.

**Methods:**

A systematic literature search was conducted in PubMed, Embase, Cochrane Library, and Web of Science up to January 31, 2026, to identify studies evaluating intraoperative ICG fluorescence imaging in patients undergoing MIE for EC. Perioperative outcomes were independently extracted by two reviewers and synthesized using a random-effects model. Odds ratios (ORs) and mean differences (MDs) with 95% confidence intervals (CIs) served as the summary statistics. Subgroup analyses and sensitivity analyses were subsequently performed.

**Results:**

A total of eight retrospective cohort studies with 1,314 patients were included. Patients were classified into the ICG fluorescence imaging group and the control group. The analysis demonstrated that ICG fluorescence imaging was associated with a significantly lower risk of AL (OR = 0.37; 95% CI: 0.18 to 0.76; *p* = 0.007). There were no significant between-group differences in operative time, intraoperative blood loss, cardiovascular complications, or postoperative length of stay (LOS), with a trend toward reduced postoperative pneumonia observed in the ICG group. Subgroup analyses revealed that the protective effect of ICG against AL was more pronounced in patients undergoing McKeown esophagectomy (OR = 0.31; 95% CI: 0.13 to 0.75; *p* = 0.009) and those with body mass index (BMI) <24 (OR = 0.35; 95% CI: 0.13 to 0.94; *p* = 0.04). Sensitivity analysis validated the reliability of the primary result on AL.

**Conclusion:**

Intraoperative ICG fluorescence imaging is significantly associated with a lower risk of AL following MIE for EC, with no significant impact on operative time, blood loss, or perioperative complications. The association between ICG application and reduced AL risk is particularly prominent in patients undergoing McKeown esophagectomy and in those with a BMI <24.

**Systematic review registration:**

https://www.crd.york.ac.uk/PROSPERO/view/CRD420261283021, identifier: CRD420261283021

## Introduction

Esophageal cancer (EC) has a high incidence and mortality worldwide and constitutes a major threat to human health (1). As a highly prevalent gastrointestinal malignancy, EC is primarily managed with a comprehensive treatment strategy centered on radical surgical resection. Specifically, radical surgery remains the cornerstone of curative-intent treatment for early-stage EC, and an indispensable component of multimodal management for locally advanced diseases (2). In recent years, the standardized implementation of minimally invasive esophagectomy (MIE), together with the widespread application of the Enhanced Recovery After Surgery (ERAS) protocol, has significantly improved perioperative care pathways and enhanced surgical safety for patients with EC (3). However, prevention and management of digestive tract reconstruction-related complications remain a core clinical challenge within the MIE (4). Among the various anastomosis-related complications, anastomotic leakage (AL) is widely recognized as a major adverse event affecting both perioperative outcomes and long-term prognosis in patients with EC (5–7). According to the Esophagectomy Complications Consensus Group (ECCG) criteria, AL is defined as a full-thickness defect at the anastomotic site, accompanied by leakage of intraluminal contents (8). This complication not only prolongs the recovery and increases medical burden but also adversely affects long-term quality of life and prognosis. Even in high-volume medical centers with advanced technical expertise, the occurrence of this complication cannot be completely eliminated.

Existing evidence has confirmed a close correlation between the risk of post-esophagectomy AL and the reconstruction approach: the McKeown esophagectomy with cervical anastomosis is associated with a significantly higher risk of leakage than the Ivor Lewis esophagectomy using intrathoracic anastomosis (9, 10). In clinical practice, multiple independent risk factors for this complication have been well characterized, spanning patient baseline characteristics, including nutritional reserve and chronic comorbid conditions, as well as technical surgical variables such as the extent of lymph node dissection (11–13). Of these identified risk factors, the regional blood perfusion of the gastric conduit is widely recognized as the key determinant of anastomotic healing (14). Therefore, accurate and objective intraoperative assessment of blood perfusion at the anastomotic site is essential for optimizing surgical decision-making and reducing the risk of AL (15).

Nevertheless, conventional intraoperative assessment relies heavily on the surgeons’ subjective judgment of visual indicators, including gastric conduit color, bleeding from the cut edge, and arterial pulsation. These methods are inherently influenced by individual expertise and intraoperative conditions, and their lack of objective, reproducible, and quantifiable criteria hinders the accurate identification of areas with potential perfusion insufficiency (16, 17). To improve the reliability of intraoperative perfusion assessment, various monitoring techniques have been continuously developed. Although modalities such as Doppler ultrasound and spectral imaging have been explored, their clinical adoption remains limited due to challenges related to operational stability, consistency of interpretation, or insufficient correlation with clinical outcomes (18, 19). In contrast, indocyanine green (ICG) near-infrared fluorescence angiography enables real-time intraoperative visualization of tissue microcirculation perfusion after intravenous administration of the fluorescent tracer (15). This technique provides an intuitive, dynamic assessment of anastomotic site perfusion, equipping surgeons with objective data to guide critical intraoperative decisions, including the timely adjustment of the anastomotic location or reconstructive strategy—to correct hypoperfusion at its source and reduce the risk of impaired anastomotic healing (15, 16).

Although ICG based fluorescence imaging technology has accumulated considerable clinical experience in liver (20, 21), gastric (22, 23), and pulmonary surgeries (24), high-level evidence supporting its role in preventing AL during MIE for EC remains limited. Existing studies have reported heterogeneous findings, and a consensus on its widespread clinical adoption is still lacking. In this context, the present study aims to conduct a systematic review and meta-analysis to rigorously synthesize available clinical data and objectively evaluate the efficacy of intraoperative ICG fluorescence imaging in preventing AL after esophagectomy for EC. The aim is to provide reliable scientific evidence to support the consistent use and personalized selection of this technology, thereby helping to improve precision and safety in esophageal surgery.

## Materials and methods

This systematic review and meta-analysis was designed and reported in accordance with the PRISMA Statement and the MOOSE Guidelines (25, 26). The study protocol has been registered in PROSPERO (International Prospective Register of Systematic Reviews)^1^ with the registration number CRD420261283021.

### Databases and search strategy

A comprehensive literature search was performed across four online databases: PubMed, Embase, the Cochrane Library, and Web of Science, with the search period updated to January 31, 2026. The search strategy combined Medical Subject Headings (MeSH terms) and free-text keywords. The main search terms included “Anastomotic leakage” and “Indocyanine Green,” which were combined using Boolean operators (AND, OR). The detailed search strategies for each database are presented in Supplementary Table 1.

Literature screening was conducted independently by two investigators (Y-hL and J-hQ), and the results were cross-checked. A backward reference search was also performed on the excluded literature to identify potentially missed relevant studies. Any discrepancies during the screening process were resolved through discussion until consensus was reached.

### Study selection and criteria

The inclusion and exclusion criteria of this study were defined as follows:

#### Inclusion criteria

(1) Adult patients (aged ≥18 years) who underwent MIE; (2) Pathologically confirmed primary EC; (3) Intraoperative use of ICG fluorescence imaging for the assessment of gastric conduit perfusion; (4) Study design: randomized controlled trial (RCT) or cohort study; (5) At least one prespecified outcome measure of this meta-analysis was reported.

#### Exclusion criteria

(1) Non-primary EC, including gastroesophageal junction cancer, gastric cancer, or other upper gastrointestinal tract tumors; (2) Open esophagectomy or other non-minimally invasive surgical approaches; (3) ICG fluorescence imaging was not used in the experimental group, or its application method was not clearly described; (4) Case reports, reviews, meta-analyses, conference abstracts, letters, editorials, or non-controlled studies; (5) No relevant outcome measures were reported; (6) Non-human studies, including animal experiments and ex vivo studies; (7) Publications not in English or Chinese; (8) For duplicate publications from the same dataset, only the most comprehensive report was included.

### Surgical procedures

In all studies included in this meta-analysis, the surgical procedures performed on patients were either the Ivor Lewis or McKeown approach. Both are classic radical esophagectomy procedures for EC, and both adopt a right transthoracic approach. The Ivor Lewis approach involves gastric mobilization via a right thoracotomy combined with a midline upper abdominal incision, followed by elevation of the gastric tube into the thoracic cavity to perform intrathoracic esophagogastric anastomosis below the level of the aortic arch. The McKeown approach involves gastric tube creation via a right thoracotomy and a midline upper abdominal incision, with an additional cervical incision along the anterior border of the left sternocleidomastoid muscle; the gastric tube is then elevated above the level of the thoracic inlet in the neck to complete cervical esophagogastric anastomosis (27). Both of the above procedures can now be performed via either classic open surgery or a combined thoracoscopic-laparoscopic approach.

### ICG fluorescence imaging protocol

After completion of gastric tube construction and prior to elevation of the gastric tube for esophagogastric anastomosis, the general operational procedure of ICG fluorescence imaging across included studies was as follows: a rapid intravenous bolus injection of ICG (dose range: 2.5–25 mg) was administered via either central or peripheral venous access, immediately followed by a rapid flush with 5–10 mL normal saline to ensure complete delivery of the full dose into the systemic circulation. At 15–30 s after ICG administration, the near-infrared fluorescence imaging system was activated to dynamically acquire real-time fluorescence signals of the gastric wall tissue, with a standardized continuous observation duration of 1–3 min. All perfusion assessments were completed within the validated 5 min effective fluorescence imaging window after ICG administration, for the objective evaluation of full-thickness gastric wall blood perfusion.

For anastomotic site selection, all included studies adopted the same core principle: the anastomosis was required to be created at the gastric wall region with confirmed adequate blood perfusion by fluorescence imaging within 5 min after ICG administration, with core diagnostic criteria for adequate perfusion including rapid onset of fluorescence signal, homogeneous full-thickness distribution, complete and persistently stable fluorescence filling; regions with delayed fluorescence onset, inhomogeneous distribution, filling defect, or absent fluorescence signal indicating hypoperfusion or non-perfusion must be strictly avoided.

### Endpoints and outcome measures

The primary outcome measure of this meta-analysis was AL following MIE for EC. For this meta-analysis, AL was defined in strict accordance with the standard definition proposed by the ECCG: a full-thickness gastrointestinal defect involving the esophagus, anastomosis, staple line, or gastric conduit, irrespective of clinical presentation or diagnostic method. This definition is currently the most widely accepted international consensus for standardized reporting of AL after esophagectomy, enabling consistent cross-study comparison (8, 28).

The secondary outcome measures included: (1) Operative time: Total duration from skin incision to completion of wound closure; (2) Intraoperative blood loss: Total volume of blood lost during the surgical procedure; (3) Cardiovascular complications: New-onset or exacerbated cardiovascular diseases/events during the perioperative period, including but not limited to myocardial injury/myocardial infarction, arrhythmia, heart failure, and cardiac arrest; (4) Postoperative pneumonia: Newly developed pulmonary infection following surgery, typically defined as pneumonia occurring more than 48 h postoperatively and meeting clinical diagnostic criteria; (5) Postoperative length of stay (LOS): Total number of days from the completion of surgery to the patient meeting discharge criteria and being formally discharged from the hospital.

### Data collection

Two independent investigators (Y-hL and J-hQ) performed literature screening and data extraction using a pre-designed data extraction form. Any discrepancies encountered during the screening or extraction process were resolved via discussion to reach a consensus.

The following information was extracted from each included study: (1) Publication details: first author’s name, year of publication, and country; (2) Study design characteristics: study type, study period, and surgical approach; (3) Demographic data: sample size, age, gender, and comorbidities; (4) Outcome measures: operative time, intraoperative blood loss, postoperative LOS, and specific postoperative complications. No attempts were made to contact the corresponding authors for unpublished data.

### Quality assessment

Risk of bias assessment for included non-randomized cohort studies was performed using the Risk Of Bias In Non-randomized Studies—of Interventions (ROBINS-I) tool, the recommended standard tool for non-randomized intervention studies by the Cochrane Handbook for Systematic Reviews of Interventions (29). We assessed the risk of bias across seven pre-specified core domains: confounding, selection of participants, classification of interventions, deviations from intended interventions, missing data, measurement of outcomes, and selective reporting of results. The overall risk of bias for each study was graded as low, moderate, serious, critical, or no information. Meanwhile, the Newcastle-Ottawa Scale (NOS) was used for supplementary quality assessment of included cohort studies (30). All included studies had an overall NOS score ≥6, indicating acceptable methodological quality for supplementary reference.

Given the specificity of ICG fluorescence imaging as an intraoperative intervention, blinding patients and surgical teams is generally not feasible in clinical practice. Therefore, studies that did not explicitly report the implementation of blinding were presumed to have a high risk of performance bias.

The quality assessment of the included studies was independently performed by the same two investigators (Y-hL, J-hQ). Any discrepancies were addressed through discussion until a consensus was achieved.

### Statistical analysis

The odds ratio (OR) with a 95% confidence interval (CI) was used as the pooled effect size metric for dichotomous variables. The mean difference (MD) with 95% CI was adopted for continuous variables. Studies that did not report the standard deviation (SD) were excluded from quantitative synthesis.

In accordance with the Cochrane Handbook for Systematic Reviews of Interventions, reasonable estimation of missing SDs was only performed when the included studies had adequate sample sizes and the outcome measures followed a normal distribution (31).

Heterogeneity across studies was assessed using the Cochrane *Q* test and *I*^2^ statistic. Substantial heterogeneity was defined as *I*^2^ > 50%. Two-sided tests were used for all analyses, and *p* < 0.05 was considered statistically significant. A random-effects model was applied to pool effect sizes. Publication bias was evaluated using Egger’s test, with *p* < 0.05 indicating significant publication bias. Sensitivity analyses were conducted using the leave-one-out method to examine the influence of each study on the stability of pooled effect sizes. All statistical analyses were performed using Review Manager (version 5.4) and Stata (version 17.0).

## Results

### Literature search

**Figure 1:**
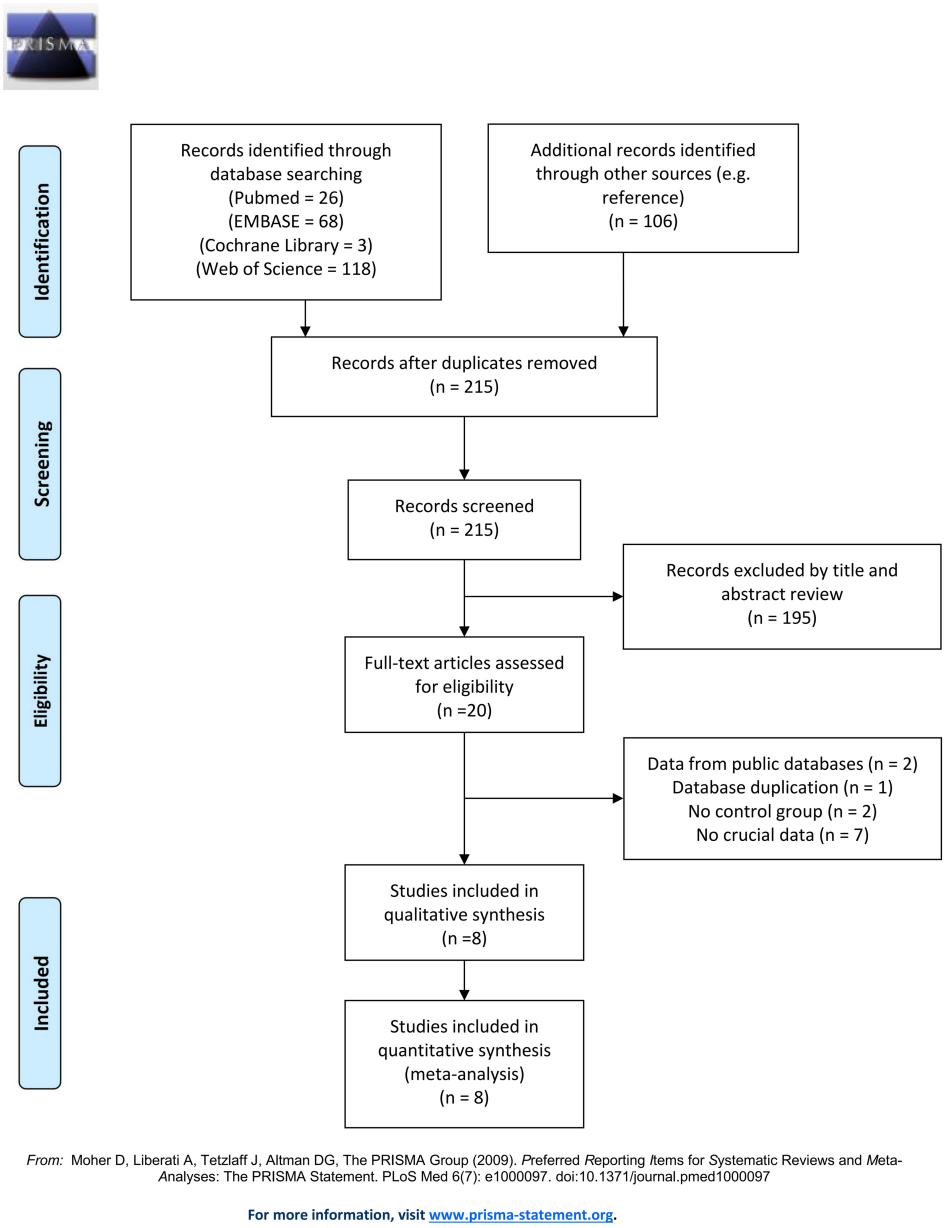
PRISMA flow diagram of literature retrieval. PRISMA, Preferred Reporting Items for Systematic Reviews and Meta-Analyses.

The literature selection process is illustrated in Figure 1. A total of 215 studies were identified through initial database searches: 26 from PubMed, 68 from Embase, 3 from the Cochrane Library, and 118 from Web of Science. An additional 106 records were obtained via manual reference checking and supplementary searches in Chinese databases. After duplicate removal and stepwise screening of titles, abstracts, and full texts, eight studies were finally included in this systematic meta-analysis (32–39).

### Characteristics of the included studies

Table 1 summarizes the baseline characteristics of the included studies. A total of eight retrospective cohort studies (RCSs) were ultimately included, with data originating from the United States, Vietnam, Belgium, Germany, Japan, and China, with a study period from 2000 to 2023. The total sample size comprised 1,314 patients, with 512 in the intervention group (Group T) and 802 in the control group (Group C). The mean age of patients ranged from 60 to 67 years, with a male predominance (1,067 patients, 81.2%). The primary surgical approaches adopted in the included studies were McKeown esophagectomy and Ivor Lewis esophagectomy. Reported body mass index (BMI) data revealed similar mean values between the intervention and control groups.

**Table 1:** Baseline characteristics and lifestyle factors of included studies.

Study (year)		Dalton et al. 2017	Nguyen et al. 2024	Van Daele et al. 2022	Karampinis et al. 2017	Noma et al. 2018	Ohi et al. 2017	Luo et al. 2021	Song et al. 2020
Country		United States	Vietnam	Belgium	Germany	Japan	Japan	China	China
Study period		2014–2016	2019–2023	2016–2020	2010–2016	2010–2016	2000–2015	2017–2019	2017–2019
Study type		RCS	RCS	RCS	RCS	RCS	RCS	RCS	RCS
No. of patients	Total	40	120	266	88	285	120	192	203
	T	20	88	115	33	71	59	86	40
	C	20	32	151	55	214	61	106	163
Age (years)	T	61.8 ± 12.8	62.5 ± 8.1	67 ± 9.2	65.7 ± 8.5	65.1 ± 8.06	NR	65.86 ± 6.54	63.6 ± 6.01
	C	66.2 ± 8	63.3 ± 8.1	65 ± 9.4	60.5 ± 8.5	64.9 ± 8.24	NR	64.94 ± 6.91	65.0 ± 5.44
Gender	Male	32	114	216	65	244	101	171	124
	Female	8	6	50	23	41	19	21	79
Surgical procedure		Ivor Lewis	McKeown	Ivor Lewis	McKeown and Ivor Lewis	McKeown	McKeown and Ivor Lewis	McKeown	McKeown
Dose of ICG		7.5 mg	5 mg	NR	7.5 mg	12.5 mg	2.5 mg	NR	25 mg
BMI (kg/m^2^)	T	26.4 ± 4.9	20.3 ± 2.9	25.8 ± 4.1	26.8 ± 4.2	22.05 ± 2.65	NR	22.62 ± 2.97	NR
	C	26.3 ± 4.1	18.9 ± 3.1	25.2 ± 4.9	25.8 ± 4.3	22.08 ± 3.29	NR	22.05 ± 3.36	NR
Pulmonary disease	T	3	5	19	NR	NR	NR	1	NR
	C	4	5	44	NR	NR	NR	5	NR
Cardiovascular disease	T	5	7	47	NR	NR	NR	5	NR
	C	3	1	67	NR	NR	NR	4	NR
Hypertension	T	NR	28	NR	NR	29	NR	30	NR
	C	NR	4	NR	NR	84	NR	31	NR
Diabetes mellitus	T	NR	11	17	NR	9	NR	4	NR
	C	NR	3	21	NR	35	NR	4	NR
Kidney disease	T	NR	7	4	NR	NR	NR	NR	NR
	C	NR	1	4	NR	NR	NR	NR	NR
Smoking	T	NR	44	44	14	NR	NR	37	NR
	C	NR	25	53	20	NR	NR	52	NR
NT	T	17	25	86	NR	42	NR	NR	NR
	C	19	8	115	NR	108	NR	NR	NR
NACT	T	NR	NR	19	25	36	NR	13	NR
	C	NR	NR	18	44	93	NR	12	NR
NCRT	T	NR	NR	67	6	6	NR	1	NR
	C	NR	NR	97	19	15	NR	0	NR

T, treatment group; C, control group; RCS, retrospective cohort study; ICG, indocyanine green; BMI, body mass index; NT, neoadjuvant therapy; NACT, neoadjuvant chemotherapy; NCRT, neoadjuvant chemoradiotherapy; NR, not reported.

Regarding baseline comorbidities and lifestyle factors, discrepancies were observed in the distribution of pulmonary diseases, cardiovascular diseases, hypertension, diabetes mellitus, renal diseases, and smoking history between the two groups. Additionally, several studies reported neoadjuvant therapy administration, including neoadjuvant therapy (NT), chemotherapy (NACT), and chemoradiotherapy (NCRT), though the proportion of patients receiving such treatments varied across studies between the intervention and control groups. Detailed perioperative outcome data are summarized in Tables 2, 3.

**Table 2:** Perioperative complications and detailed perioperative information of included studies.

Study (year)	Anastomotic leak		Cardiovascular complications		Pneumonia		Operative time (min)		Intraoperative blood loss (mL)		Postoperative LOS (day)	
	T	C	T	C	T	C	T	C	T	C	T	C
Dalton et al. 2018 (36)	2	0	5	5	NR	NR	379 ± 66	371 ± 90	338 ± 723	159 ± 150	NR	NR
Nguyen et al. 2024 (38)	27	12	2	1	16	9	376.9 ± 66.2	388.7 ± 63.3	NR	NR	11 ± 0.82	11.23 ± 0.97
Van Daele et al. 2022 (33)	17	26	NR	NR	NR	NR	NR	NR	NR	NR	NR	NR
Karampinis et al. 2017 (35)	1	10	NR	NR	NR	NR	NR	NR	NR	NR	NR	NR
Noma et al. 2018 (39)	6	54	NR	NR	12	53	596.5 ± 90.8	620.6 ± 102.5	NR	NR	24.4 ± 15.9	25.5 ± 12.7
Ohi et al. 2017 (37)	1	9	NR	NR	NR	NR	NR	NR	NR	NR	NR	NR
Luo et al. 2021 (34)	1	11	0	1	2	5	249.77 ± 80.8	242.58 ± 57.92	105.47 ± 57.12	109.91 ± 76.13	13.85 ± 10.72	16.74 ± 17.24
Song et al. 2020 (32)	1	26	NR	NR	4	17	250.47 ± 29.19	244.98 ± 33.15	171.25 ± 44.98	184.27 ± 50.40	NR	NR

LOS, length of stay; T, treatment group; C, control group; NR, not reported.

**Table 3:** Clinical information of tumors in patients of included studies.

Study (year)	Cervical and upper third		Middle third		Lower third		T_1_		T_2_		T_3_		T_4_		ADC		SCC		Except for A&S	
	T	C	T	C	T	C	T	C	T	C	T	C	T	C	T	C	T	C	T	C
Dalton et al. 2018 (36)	NR	NR	NR	NR	NR	NR	NR	NR	NR	NR	NR	NR	NR	NR	2	14	NR	NR	NR	NR
Nguyen et al. 2024 (38)	4	2	36	18	46	11	5	2	15	1	39	19	29	10	82	2	81	30	NR	NR
Van Daele et al. 2022 (33)	2	1	19	26	94	124	NR	NR	NR	NR	NR	NR	NR	NR	23	110	23	41	NR	NR
Karampinis et al. 2017 (35)	NR	NR	NR	NR	NR	NR	NR	NR	NR	NR	NR	NR	NR	NR	NR	NR	NR	NR	NR	NR
Noma et al. 2018 (39)	10	40	34	101	27	73	33	88	19	42	14	63	1	14	68	12	65	193	3	9
Ohi et al. 2017 (37)	NR	NR	NR	NR	NR	NR	NR	NR	NR	NR	NR	NR	NR	NR	NR	NR	NR	NR	NR	NR
Luo et al. 2021 (34)	2	8	35	50	49	48	20	33	20	15	46	57	0	1	84	0	84	101	0	5
Song et al. 2020 (32)	8	18	21	80	11	65	10	41	22	86	8	36	NR	NR	NR	NR	39	155	NR	NR

T, treatment group; C, control group; Tumor location: cervical and upper third, middle third, lower third; Tumor stage: T1, T2, T3, T4; ADC, adenocarcinoma; SCC, squamous cell carcinoma; A&S, adenocarcinoma and squamous cell carcinoma; NR, not reported.

### Risk of bias assessment

The primary risk of bias assessment results for the included RCSs using the ROBINS-I tool are summarized in Supplementary Tables 2, 3. All included studies met the pre-specified methodological criteria for quantitative synthesis in this meta-analysis, and were fully included in the primary pooled analysis. The supplementary NOS scoring results for all included studies are presented in Supplementary Table 4, where all 8 studies achieved a NOS score ≥6, serving only as a secondary reference for methodological quality.

### Surgical information

**Figure 2:**
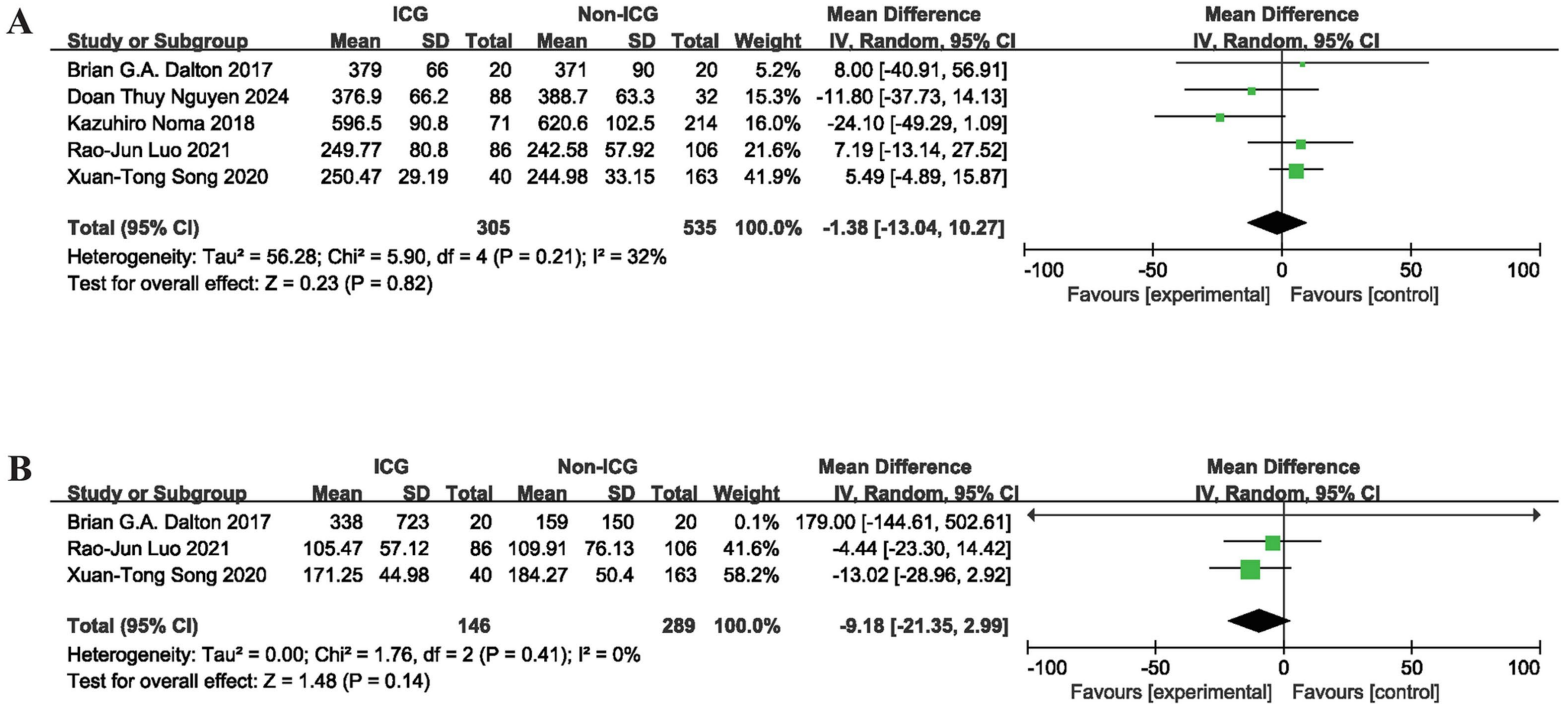
Meta-analysis of perioperative indicators in MIE: comparing the group using intraoperative ICG fluorescence imaging with the control group. **(A)** Operative time; **(B)** Intraoperative blood loss; MIE, minimally invasive esophagectomy; ICG, indocyanine green; SD, standard deviation; CI, confidence interval.

Of the eight included studies, five documented operative time, a critical perioperative outcome. The meta-analysis findings indicated that ICG fluorescence imaging did not result in a statistically significant reduction in operative time relative to the control group (MD = −1.38, 95% CI: −13.04 to 10.27; *p* = 0.82), as depicted in Figure 2A. Egger’s test revealed no evidence of publication bias (*p* = 0.700). Three studies assessed the effect of ICG fluorescence imaging on intraoperative blood loss. The results showed a tendency toward reduced intraoperative blood loss with ICG fluorescence imaging; however, this difference did not reach statistical significance (MD = −9.18, 95% CI: −21.35 to 2.99; *p* = 0.14), as illustrated in Figure 2B. Egger’s test also indicated no publication bias (*p* = 0.525).

### Postoperative complications

**Figure 3:**
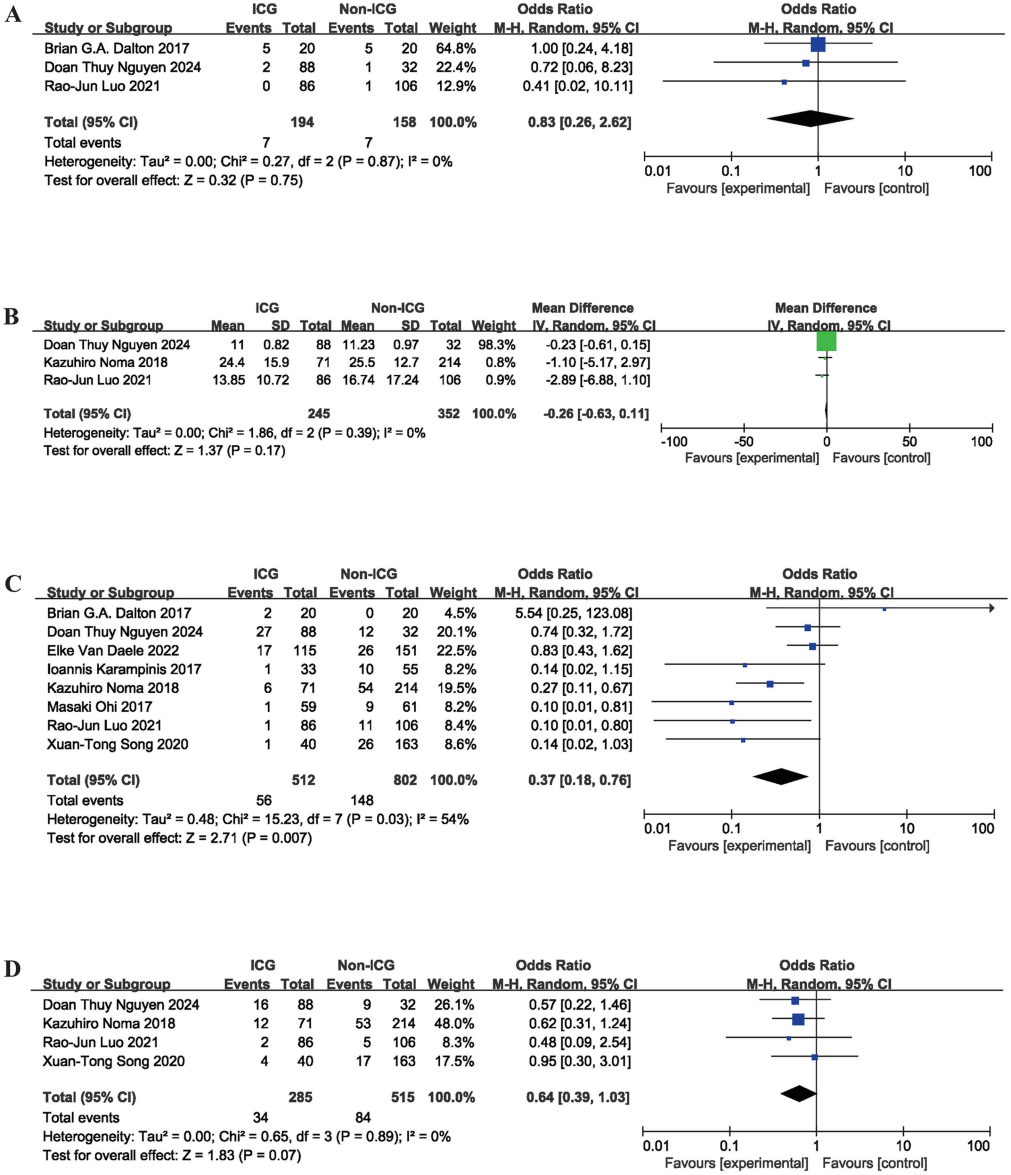
Meta-analysis of perioperative indicators in MIE: comparing the group using intraoperative ICG fluorescence imaging with the control group. **(A)** Cardiovascular complications; **(B)** Postoperative LOS; **(C)** Anastomotic Leakage; **(D)** Pneumonia; MIE, minimally invasive esophagectomy; ICG, indocyanine green; LOS, length of stay; SD, standard deviation; CI, confidence interval.

Comprehensive data on postoperative complications from the eight qualifying studies are displayed in Table 2. This meta-analysis rigorously assessed the influence of ICG fluorescence imaging on a range of postoperative complications and postoperative LOS subsequent to MIE for EC. The findings indicated that ICG fluorescence imaging did not significantly reduce the risk of postoperative cardiovascular complications (OR = 0.83, 95% CI: 0.26 to 2.62; *p* = 0.75) or shorten postoperative LOS (MD = −0.26, 95% CI: −0.63 to 0.11; *p* = 0.17), as depicted in Figures 3A,B. Egger’s tests for these two outcomes also revealed no evidence of publication bias (cardiovascular complications: *p* = 0.141; postoperative LOS: *p* = 0.411).

In contrast, the meta-analysis showed that intraoperative ICG fluorescence imaging was significantly associated with a lower incidence of AL, the core postoperative complication of MIE (OR = 0.37, 95% CI: 0.18 to 0.76; *p* = 0.007). Furthermore, albeit not achieving the conventional threshold for statistical significance, a trend toward reduced postoperative pneumonia incidence was noted with ICG fluorescence imaging (OR = 0.64, 95% CI: 0.39 to 1.03; *p* = 0.07). The findings are illustrated in Figures 3C,D. Egger’s tests for these two outcomes also showed no evidence of publication bias (AL: *p* = 0.215; pneumonia: *p* = 0.975).

**Figure 4:**
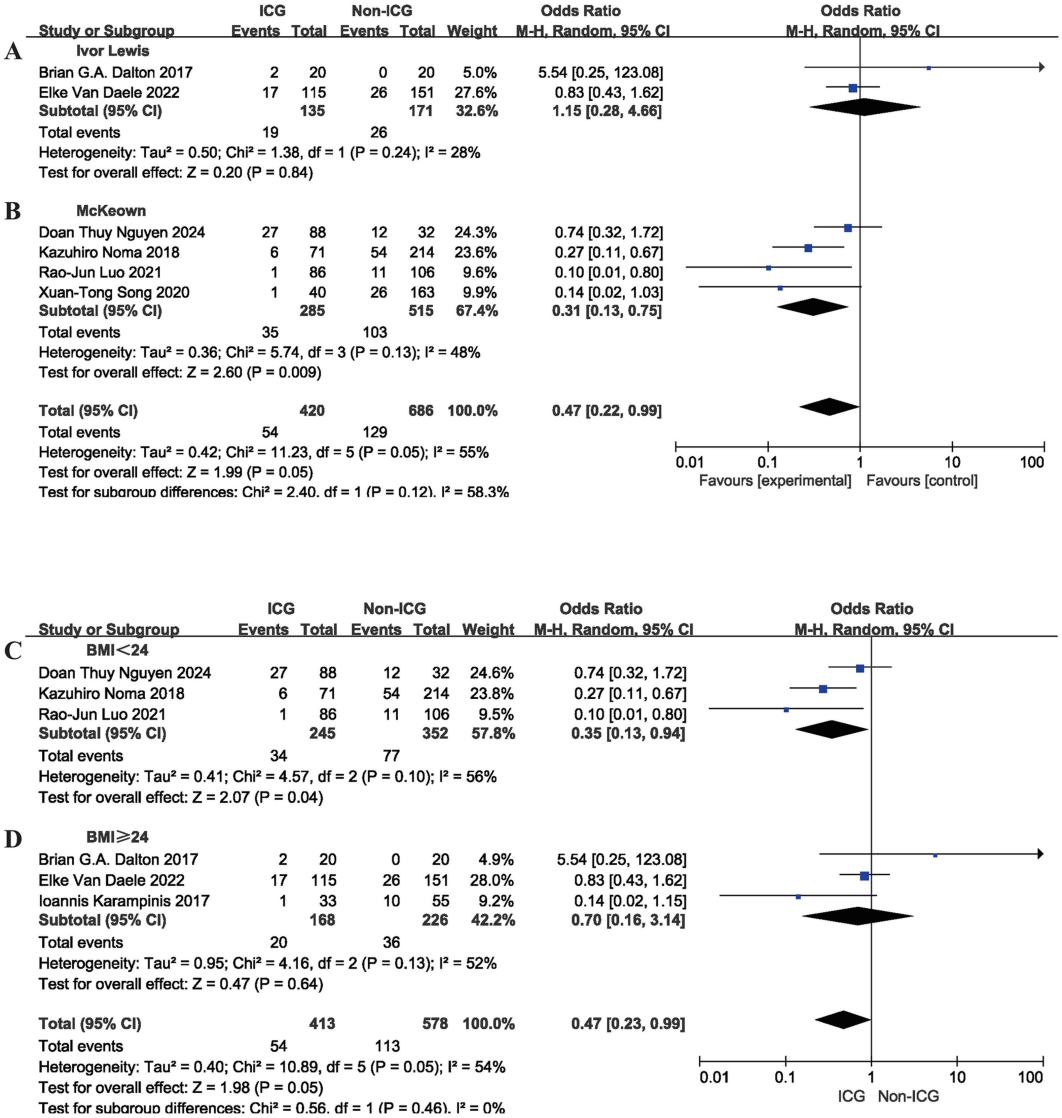
Meta-analysis of perioperative indicators in MIE: comparing the group using intraoperative ICG fluorescence imaging with the control group. **(A)** Ivor Lewis; **(B)** Mckeown; **(C)** BMI < 24; **(D)** BMI ≥ 24; MIE, minimally invasive esophagectomy; ICG, indocyanine green; BMI, body mass index; SD, standard deviation; CI, confidence interval.

Subgroup analyses were performed to further explore the association between surgical technique and the relationship between intraoperative ICG fluorescence imaging and postoperative AL risk after MIE. Stratification by surgical technique showed that no statistically significant difference was observed in patients undergoing the Ivor Lewis procedure (intrathoracic anastomosis) (OR = 1.15, 95% CI: 0.28 to 4.66; *p* = 0.84). Among patients undergoing the McKeown procedure (cervical anastomosis), ICG fluorescence imaging was significantly associated with a lower risk of postoperative AL (OR = 0.31, 95% CI: 0.13 to 0.75; *p* = 0.009), as illustrated in Figures 4A,B.

Additionally, subgroup analysis based on BMI identified another influencing factor. In patients with BMI < 24, ICG fluorescence imaging was significantly associated with a lower risk of postoperative AL (OR = 0.35, 95% CI: 0.13 to 0.94; *p* = 0.04). In patients with BMI ≥ 24, although the OR trended toward an association with reduced AL risk, the difference did not reach statistical significance (OR = 0.70, 95% CI: 0.16 to 3.14; *p* = 0.64), as illustrated in Figures 4C,D.

### Publication bias and sensitivity analysis

Visual inspection of funnel plots revealed no significant asymmetry (Supplementary Figure 1). In addition, Egger’s test showed no significant indication of publication bias among the included studies. Sensitivity analyses were performed using the leave-one-out method to evaluate the stability and reliability of the meta-analysis results. As demonstrated in Supplementary Figure 2, following the sequential exclusion of each individual study, the adjusted pooled effect sizes did not display directional changes and remained within the CI of the original estimate.

## Discussion

AL is a significant complication following MIE, directly linked to inadequate microcirculatory perfusion in the gastroesophageal anastomotic region. It remains a fundamental challenge in digestive tract reconstruction during esophageal surgery. Conventional intraoperative blood flow assessment relies exclusively on the surgeon’s subjective judgment of gastric wall color, wound exudation, and other markers, an approach that is susceptible to interference from surgical field conditions, operative experience, and other variables. Devoid of objective and quantitative evidence, this approach fails to accurately identify concealed hypoperfusion areas, rendering complete avoidance of AL challenging even in experienced medical facilities. ICG fluorescence imaging enables real-time visualization of tissue microcirculation via intravenous tracing, potentially optimizing blood supply assessment and guiding intraoperative anastomotic decisions to mitigate AL risk. Nonetheless, the genuine clinical efficacy of this technique in MIE lacks comprehensive quantitative evaluation and evidence-based validation. Adhering strictly to PRISMA guidelines, this systematic review and meta-analysis included eight high-quality retrospective cohort studies involving 1,314 EC patients who underwent MIE. As the inaugural systematic assessment of ICG fluorescence imaging for postoperative AL prevention, this study investigated its effects on perioperative outcomes and factors contributing to efficacy heterogeneity, aiming to provide evidence-based guidance for the standardized and personalized clinical implementation of this technique.

The findings of this meta-analysis demonstrate a significant association between intraoperative ICG fluorescence imaging and reduced odds of postoperative AL following MIE (OR = 0.37, 95% CI: 0.18 to 0.76; *p* = 0.007). No statistically significant differences were observed between the ICG and control groups in operative time (MD = −1.38, 95% CI: −13.04 to 10.27; *p* = 0.82), intraoperative blood loss (MD = −9.18, 95% CI: −21.35 to 2.99; *p* = 0.14), perioperative cardiovascular complications (OR = 0.83, 95% CI: 0.26 to 2.62; *p* = 0.75), or postoperative LOS (MD = −0.26, 95% CI: −0.63 to 0.11; *p* = 0.17). A favorable trend toward reduced postoperative pneumonia incidence was observed (OR = 0.64, 95% CI: 0.39 to 1.03; *p* = 0.07). Subgroup analyses identified patients who underwent the McKeown procedure (OR = 0.31, 95% CI: 0.13 to 0.75; *p* = 0.009) and those with a BMI < 24 (OR = 0.35, 95% CI: 0.13 to 0.94; *p* = 0.04) as the primary beneficiaries. Sensitivity analyses using the leave-one-out method confirmed no substantial divergence in pooled effect sizes, indicating robust study results.

The pathophysiology of AL is closely linked to inadequate microcirculatory perfusion in the anastomotic region, which is a fundamental technical challenge in digestive tract reconstruction during esophageal surgery. The observed association between ICG fluorescence imaging and lower AL risk may be primarily explained by the ability of this technique to overcome the subjective limitations of conventional intraoperative blood supply assessment. As noted earlier, conventional intraoperative evaluation is susceptible to interference and fails to detect concealed hypoperfusion, which is a key contributor to the persistent incidence of AL even in high-volume specialized centers. In contrast, ICG fluorescence imaging enables real-time visual assessment of tissue microcirculation via intravenous tracing, intuitively and dynamically illustrating the blood perfusion distribution in the tubular stomach and anastomotic region. This allows surgeons to promptly adjust anastomotic sites or reconstruction strategies intraoperatively, avoiding anastomosis in poorly perfused areas and mitigating AL risk at its source. This finding aligns with the application of ICG fluorescence imaging in gastrointestinal, hepatobiliary, and other surgical fields, affirming that precise intraoperative blood supply assessment is critical for improving the healing quality of digestive tract anastomoses.

Therefore, the core intraoperative application principle of ICG fluorescence imaging in clinical esophageal surgery is straightforward and actionable. For a planned anastomotic region with confirmed high perfusion on ICG imaging, direct anastomosis is performed. In cases where the target region demonstrates hypoperfusion, the ischemic segment of the gastric tube is first revised or resected, followed by anastomosis reconstruction at a newly identified, high-perfusion gastric wall site. This perfusion-guided anastomotic site selection strategy may avoid forced anastomosis in poorly perfused segments, which could in turn reduce the theoretical risk of impaired anastomotic healing and AL.

Furthermore, previous studies have well established that malnutrition is an independent risk factor for elevated risk of postoperative complications and mortality in patients with malignancy (40). In patients with EC, due to factors including dysphagia and obstruction caused by the primary tumor and the hypermetabolic state of the malignancy, the preoperative incidence of malnutrition is significantly higher than that in patients with other gastrointestinal malignancies, and malnutrition is also an independent high-risk factor for the development of AL. Accordingly, preoperative nutritional status should be regarded as one of the key indicators for AL risk assessment. For patients at high risk of severe malnutrition, nutritional support should be administered in accordance with clinical guideline recommendations during the perioperative period, with enteral nutrition as the first-line option and parenteral nutrition supplemented when necessary, to improve the patients’ nutritional reserves and reduce the risk of poor anastomotic healing.

In addition, of the eight studies included in this meta-analysis, at least half documented a definitive smoking history in the enrolled patients, and smoking has been confirmed as a critical risk factor for esophageal AL (41). Smoking impairs tissue microcirculation and weakens tissue healing capacity (42), thereby compromising the accuracy of tissue perfusion assessment via ICG fluorescence imaging. To reduce the incidence of AL after esophagectomy for EC, implementation of a structured preoperative smoking cessation program with a minimum duration of 4 weeks is warranted in patients who smoke (43–45).

The observation that ICG fluorescence imaging does not substantially influence operative time or intraoperative blood loss aligns with clinical practice logic. Characterized by simplicity, this technique only requires intravenous administration of a contrast agent followed by real-time imaging via near-infrared equipment—no additional invasive procedures or surgical steps that would disrupt the existing workflow. Theoretically, this should not prolong operative time. Moreover, as an exclusively intraoperative assessment tool, it does not alter core procedures such as tumor resection, lymph node dissection, or digestive tract reconstruction, thus exerting no significant impact on intraoperative blood loss. These data further indicate that ICG fluorescence imaging does not increase intraoperative trauma or procedural burden during esophageal surgery. The observed association between ICG use and reduced AL risk, without additional operative burden, aligns well with the core principles of the ERAS paradigm, supporting its potential for broader clinical application. Regarding the favorable yet non-significant trend toward reduced postoperative pneumonia (*p* = 0.07), it is hypothesized that this trend may be attributed to fewer AL-related secondary thoracic infections and mitigation of perioperative inflammatory responses, which could positively influence pulmonary outcomes. Nevertheless, the restricted sample size, together with the multifactorial characteristics of pneumonia, may have undermined statistical power. Further large-scale studies are needed to confirm this potential advantage.

Subgroup analyses revealed considerable heterogeneity in the strength of the association between ICG use and reduced AL risk across patient populations, with surgical approach and BMI identified as key factors influencing this association. In the subgroup stratified by surgical approach, a significant negative association between ICG fluorescence imaging and AL risk was observed in patients undergoing the McKeown procedure (OR = 0.31, 95% CI: 0.13 to 0.75; *p* = 0.009), while no significant association was detected in those undergoing the Ivor Lewis procedure (OR = 1.15, 95% CI: 0.28 to 4.66; *p* = 0.84). This difference may be primarily attributed to the characteristics of cervical anastomosis in the McKeown procedure: this approach requires traction of the gastric conduit through the mediastinum or retrosternal space to the neck, a maneuver that may easily cause vascular traction and torsion, thus impairing blood perfusion of the distal gastric conduit. Additionally, the cervical region has inherently poorer blood supply than the intrathoracic cavity, resulting in a higher AL risk than that of the intrathoracic anastomosis in the Ivor Lewis procedure. The objective blood supply assessment provided by ICG fluorescence imaging may help avoid anastomosis in hypoperfused areas, which may explain the more pronounced association with reduced AL risk observed in this subgroup. In contrast, the Ivor Lewis procedure involves intrathoracic anastomosis without long-distance traction of the tubular stomach, resulting in a more stable blood supply and lower baseline AL risk-which may have diluted the observable association between ICG use and AL risk reduction. Furthermore, the relatively small sample size of Ivor Lewis cases in the included studies may have contributed to insufficient statistical power, another potential reason for the non-significant result. In the subgroup stratified by BMI, an association between ICG fluorescence imaging and reduced AL risk was observed in patients with BMI < 24 (OR = 0.35, 95% CI: 0.13 to 0.94; *p* = 0.04) but not in those with BMI ≥ 24 kg/m^2^ (OR = 0.70, 95% CI: 0.16 to 3.14; *p* = 0.64). This difference is closely linked to the characteristics of near-infrared fluorescence imaging: patients with elevated BMI often have excessive thoracic and abdominal adipose tissue, obstructing near-infrared light penetration and imaging clarity, reducing the accuracy of microcirculatory perfusion assessment. Additionally, these patients are predisposed to vascular endothelial dysfunction and metabolic disorders, which may impair tubular stomach blood supply and weaken the intervention effect of ICG fluorescence imaging. In contrast, patients with lower BMI have clearer surgical fields, which may support more accurate ICG fluorescence imaging assessment, corresponding to the more pronounced association with reduced AL risk observed in this subgroup.

Among the primary studies included in this meta-analysis, a proportion of enrolled patients received preoperative neoadjuvant chemotherapy or neoadjuvant chemoradiotherapy. Regarding the correlation between neoadjuvant therapy and AL, current evidence-based data demonstrate that there is no statistically significant difference in the incidence of AL between patients receiving preoperative neoadjuvant therapy and those undergoing upfront surgery, with some studies even indicating a lower incidence of AL in the neoadjuvant therapy group (46, 47). Conventional wisdom holds that preoperative neoadjuvant therapy impairs local tissue blood supply and compromises tissue repair capacity, thereby increasing the risk of AL. However, a high-quality clinical study has confirmed that after strictly controlling for key confounding factors, including baseline tumor stage and patient nutritional status, the adverse impact of neoadjuvant therapy on anastomotic healing after esophagectomy for EC is not significant (46). For example, preoperative sarcopenia, as the core objective marker of malnutrition, is an independent risk factor for the development of AL in patients not receiving neoadjuvant therapy; however, this correlation has not been clearly validated in patients receiving neoadjuvant therapy (48). We hypothesize that this discrepancy may be attributed to more standardized perioperative nutritional management and more proactive nutritional status intervention in patients undergoing neoadjuvant therapy. This further suggests that, compared with neoadjuvant therapy itself, patients’ baseline physiological reserve and intraoperative anastomotic technique are more critical determinants of AL risk. Furthermore, continuous innovation in gastrointestinal anastomosis techniques, the popularization of intraoperative precise quality control technologies such as ICG fluorescence perfusion assessment, and the optimization of full-process perioperative management under the ERAS concept have all effectively offset the potential adverse effects of neoadjuvant therapy on anastomotic healing.

This study possesses several methodological strengths that enhance the credibility of its conclusions. First, it was conducted in strict compliance with PRISMA guidelines, with systematic searches across multiple databases supplemented by manual retrieval to minimize literature omission. Second, all included studies were evaluated using the ROBINS-I and NOS, and all eight achieved acceptable quality—ensuring data reliability at the source. Third, a random-effects model was employed to pool effect sizes, which addressed inter-study heterogeneity and effectively mitigated bias. No substantial publication bias was identified for any outcome measure via Egger’s test. Fourth, focusing on mainstream surgical approaches for EC, this study included only adult patients with pathologically confirmed EC, excluding gastroesophageal junction cancer and open surgery to avoid confounding factors—enhancing the clinical relevance and practicality of the findings. Fifth, subgroup analyses, sensitivity analyses, and publication bias assessments were conducted to investigate factors influencing the efficacy of this technique and verify result robustness, thereby providing detailed references for personalized clinical application.

This study also has several limitations that must be objectively acknowledged and addressed in future research. First, all included studies were retrospective cohort studies, which inherently carry selection bias and confounding bias. Unadjusted factors such as surgeon expertise, perioperative management protocols, and baseline nutritional status may have influenced the results. Additionally, the operational characteristics of ICG fluorescence imaging precluded blinding in all studies, leading to potential performance bias. Second, the overall sample size of this study is relatively limited, with only 1,314 patients included. Particularly in specific subgroups such as the Ivor Lewis esophagectomy and BMI ≥ 24, the sample size is even smaller. This may have compromised the statistical power of some results, thereby making it difficult to detect potential clinical benefits. Third, a certain degree of heterogeneity existed among the included studies. These studies were conducted across different countries and regions, including the United States, Vietnam, Belgium, Germany, Japan, and China. Notably, inter-center variations existed in the contrast agent dosage, injection timing, and imaging interpretation criteria for ICG fluorescence imaging. Meanwhile, differences were also observed in the surgical procedures of MIE and the perioperative ERAS management protocols. These factors may have exerted a certain impact on the study results. Fourth, due to the considerable heterogeneity in ICG dosage, injection timing, and perfusion interpretation criteria across included studies, this meta-analysis was unable to evaluate the influence of variable ICG fluorescence imaging parameters on the observed association between ICG use and reduced AL risk. Fifth, this study only focused on perioperative short-term outcomes and did not evaluate long-term prognostic indicators, including anastomotic stricture, long-term quality of life, and tumor recurrence or metastasis. Thus, comprehensive evaluation of the potential value of this technique in the long-term postoperative rehabilitation of patients with EC is challenging.

Based on the limitations of this study and the characteristics of current research data, future research should focus on the following directions. First, we need large-scale, multicenter, prospective RCTs—the gold standard for validating the efficacy of ICG fluorescence imaging. These studies will significantly reduce bias and provide higher-level evidence-based support for clinical application. Second, standardized protocols for the use of this technique in MIE should be established. Targeted studies are required to determine the optimal contrast agent dosage, injection timing, and imaging interpretation criteria, thereby enabling standardized application and improving result consistency across centers. Third, the sample size should be expanded to conduct in-depth subgroup analyses, further investigating the impact of neoadjuvant therapy, patient comorbidities, surgeon experience, and other factors on the efficacy of this technique. When combined with the previously identified influencing factors (surgical approach and BMI), this will clarify the optimal beneficiary population. Fourth, long-term follow-up studies should be undertaken to evaluate the impact of ICG fluorescence imaging on long-term postoperative complications, quality of life, and tumor prognosis in EC patients, thus providing a comprehensive assessment of its clinical value.

## Conclusion

This systematic review and meta-analysis comprehensively synthesized the available evidence regarding intraoperative ICG fluorescence imaging in patients undergoing MIE for EC. The findings show that ICG fluorescence imaging is significantly associated with a lower risk of postoperative AL, with no significant differences observed in key perioperative outcomes including operative time, intraoperative blood loss, incidence of postoperative cardiovascular complications, and postoperative LOS between the ICG and control groups. Additionally, a non-significant trend toward a lower incidence of postoperative pneumonia was observed in the ICG group. Subgroup analyses elucidated that the association between ICG use and reduced AL risk was more pronounced in patients undergoing the McKeown procedure and those with a BMI < 24. Distinguished by its simplicity, non-invasiveness, cost-effectiveness, and lack of additional surgical burden, the intraoperative ICG fluorescence imaging technique is suitable for promotion in medical centers at all levels performing MIE for EC. Its integration with the ERAS concept may further refine the perioperative management pathway for EC patients, with significant clinical implications for improving surgical safety and enhancing the quality of postoperative rehabilitation.

## Data Availability

The raw data supporting the conclusions of this article will be made available by the authors, without undue reservation.

## Footnotes

1
https://www.crd.york.ac.uk/PROSPERO/view/CRD420261283021
