# Supplementary figures and images for: The effectiveness of intraoperative indocyanine green fluorescence imaging in preventing anastomotic leakage after minimally invasive esophagectomy for esophageal cancer: a systematic review and meta-analysis
Source: Front Med (Lausanne). 2026 May 13;13:1830155. doi: 10.3389/fmed.2026.1830155 (PMC13213865; doi:10.3389/fmed.2026.1830155)

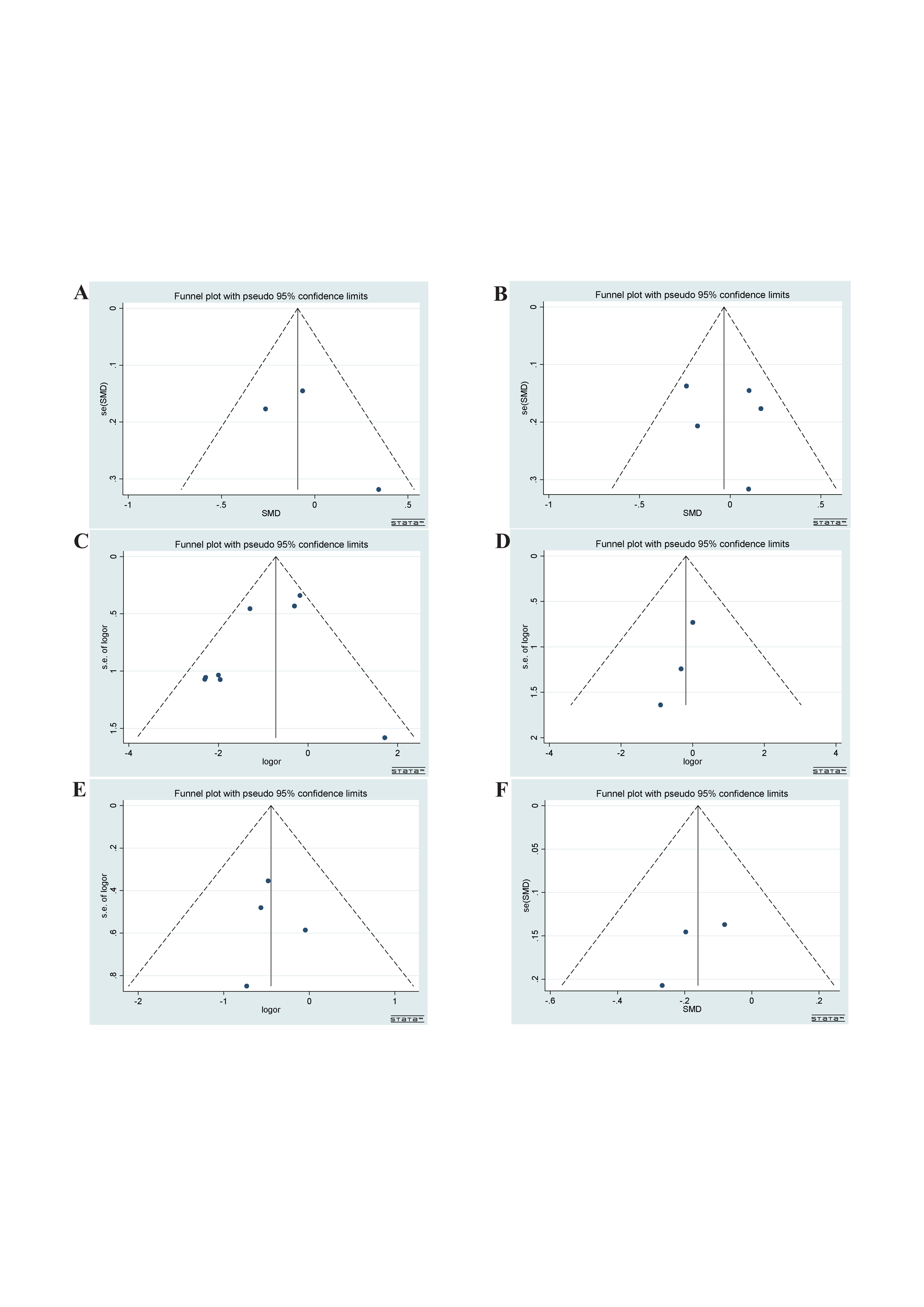

Supplement: Supplementary file 1 [file Image_1.JPEG]

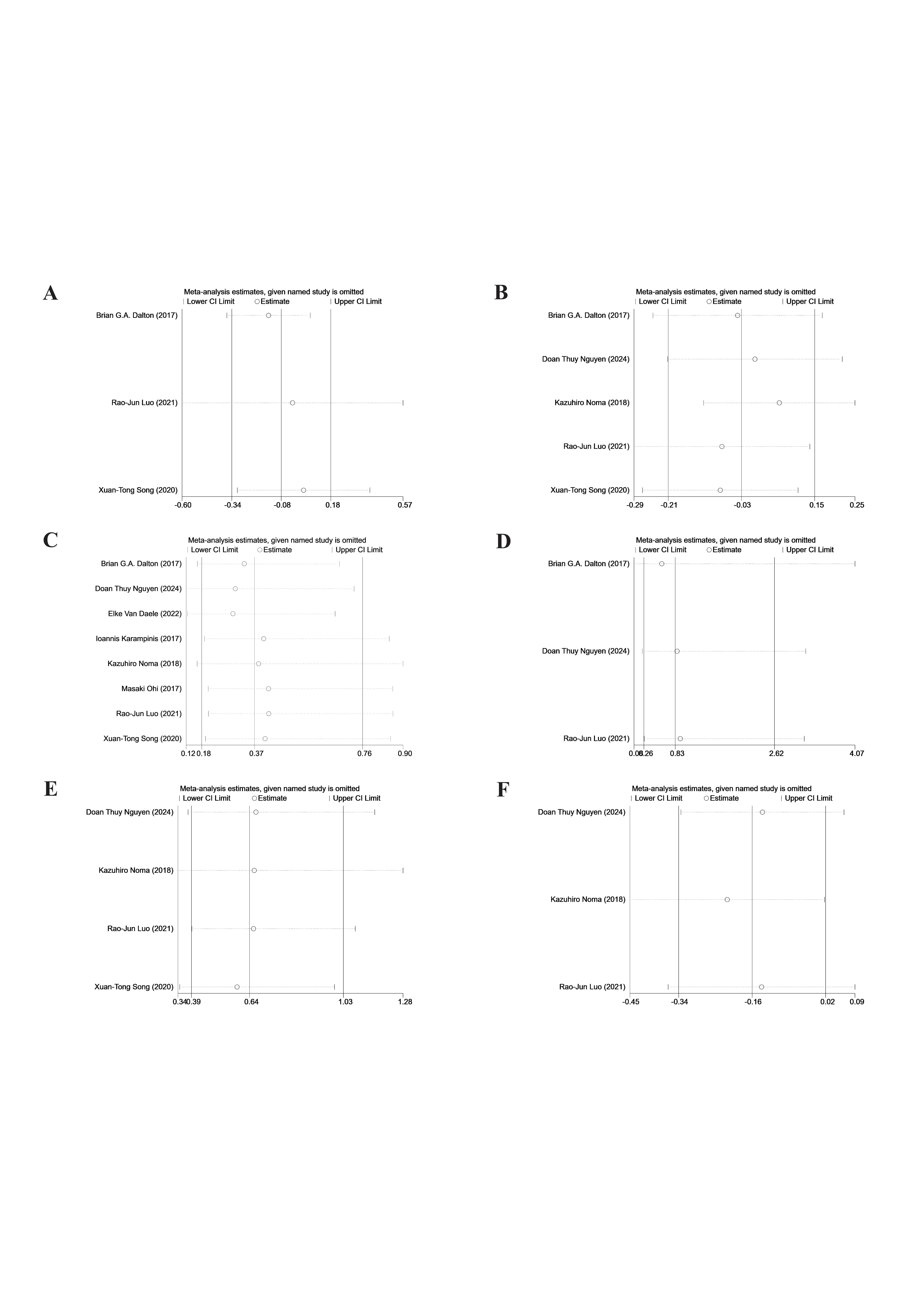

Supplement: Supplementary file 2 [file Image_2.JPEG]
