# Supplementary material for: The effectiveness of intraoperative indocyanine green fluorescence imaging in preventing anastomotic leakage after minimally invasive esophagectomy for esophageal cancer: a systematic review and meta-analysis
Source: Front Med (Lausanne). 2026 May 13;13:1830155. doi: 10.3389/fmed.2026.1830155 (PMC13213865; doi:10.3389/fmed.2026.1830155)
Supplement: Supplementary file 3 [file Supplementary_file_1.DOCX]

**Supplementary Figure Legends**

**Supplementary Figure 1.** Funnel Plots to detect publication bias. (A) Operative time; (B) Intraoperative blood loss; (C) Anastomotic Leakage; (D) Cardiovascular complications; (E) Pneumonia; (F) Postoperative LOS; LOS, length of stay.

**Supplementary Figure 2.** Sensitivity analyses of outcomes. (A) Operative time; (B) Intraoperative blood loss; (C) Anastomotic Leakage; (D) Cardiovascular complications; (E) Pneumonia; (F) Postoperative LOS; LOS, length of stay; CI, confidence interval.
