# Supplementary material for: The effectiveness of intraoperative indocyanine green fluorescence imaging in preventing anastomotic leakage after minimally invasive esophagectomy for esophageal cancer: a systematic review and meta-analysis
Source: Front Med (Lausanne). 2026 May 13;13:1830155. doi: 10.3389/fmed.2026.1830155 (PMC13213865; doi:10.3389/fmed.2026.1830155)
Supplement: Supplementary file 4 [file Table_1.DOCX]

**Supplementary Table 1.** Details of search strategy for all databases.

| **Database** | **Search Strategy** |
| --- | --- |
| Pubmed | (((((((esophageal anastomotic leak) OR (anastomotic leakage)) OR (Esophageal anastomotic fistula)) OR (Leakage of esophageal anastomosis)) OR (Anastomotic leakage in the esophagus)) OR (Esophageal anastomosis leak)) AND (((((((((((minimally invasive esophagectomy) OR (Minimal access esophagectomy)) OR (Laparoscopic assisted esophagectomy)) OR (Thoracoscopic assisted esophagectomy)) OR (Video assisted minimally invasive esophagectomy)) OR (VAMIE)) OR (McKeown esophagectomy)) OR (Ivor Lewis esophagectomy)) OR (minimally invasive Ivor Lewis esophagectomy)) OR (RAMIE)) OR (Robotic-assisted Minimally Invasive Esophagectomy))) AND ((((((((Indocyanine green) OR (Indocyanine Green Dye)) OR (Indocyanine Green for Injection)) OR (ICG)) OR (ICG fluorescence imaging)) OR (fluorescence imaging)) OR (fluorescence imaging)) OR (indocyanine green fluorescence)) |
| Embase | ('esophageal anastomotic leak' OR 'anastomotic leakage'/exp OR 'anastomotic leakage' OR 'esophageal anastomotic fistula' OR 'leakage of esophageal anastomosis' OR 'anastomotic leakage in the esophagus' OR 'esophageal anastomosis leak') AND ('minimally invasive esophagectomy' OR 'minimal access esophagectomy' OR 'laparoscopic assisted esophagectomy' OR 'thoracoscopic assisted esophagectomy' OR 'video assisted minimally invasive esophagectomy' OR 'vamie' OR 'mckeown esophagectomy' OR 'ivor lewis esophagectomy' OR 'minimally invasive ivor lewis esophagectomy' OR 'ramie' OR 'robotic-assisted minimally invasive esophagectomy') AND ('indocyanine green' OR 'indocyanine green dye' OR 'indocyanine green for injection' OR 'icg' OR 'icg fluorescence imaging' OR 'fluorescence imaging' OR 'indocyanine green fluorescence') |
| Cochrane Library | ("esophageal anastomotic leak" OR "anastomotic leakage" OR "anastomotic leakage" OR "esophageal anastomotic fistula" OR "leakage of esophageal anastomosis" OR "anastomotic leakage in the esophagus" OR "esophageal anastomosis leak") AND ("minimally invasive esophagectomy" OR "minimal access esophagectomy" OR "laparoscopic assisted esophagectomy" OR "thoracoscopic assisted esophagectomy" OR "video assisted minimally invasive esophagectomy" OR "vamie" OR "mckeown esophagectomy" OR "ivor lewis esophagectomy" OR "minimally invasive ivor lewis esophagectomy" OR "ramie" OR "robotic-assisted minimally invasive esophagectomy") AND ("indocyanine green" OR "indocyanine green dye" OR "indocyanine green for injection" OR "icg" OR "icg fluorescence imaging" OR "fluorescence imaging" OR "indocyanine green fluorescence") |
| Web of Science | ("esophageal anastomotic leak" OR "anastomotic leakage" OR "anastomotic leakage" OR "esophageal anastomotic fistula" OR "leakage of esophageal anastomosis" OR "anastomotic leakage in the esophagus" OR "esophageal anastomosis leak") AND ("minimally invasive esophagectomy" OR "minimal access esophagectomy" OR "laparoscopic assisted esophagectomy" OR "thoracoscopic assisted esophagectomy" OR "video assisted minimally invasive esophagectomy" OR "vamie" OR "mckeown esophagectomy" OR "ivor lewis esophagectomy" OR "minimally invasive ivor lewis esophagectomy" OR "ramie" OR "robotic-assisted minimally invasive esophagectomy") AND ("indocyanine green" OR "indocyanine green dye" OR "indocyanine green for injection" OR "icg" OR "icg fluorescence imaging" OR "fluorescence imaging" OR "indocyanine green fluorescence") |
