# Supplementary material for: The effectiveness of intraoperative indocyanine green fluorescence imaging in preventing anastomotic leakage after minimally invasive esophagectomy for esophageal cancer: a systematic review and meta-analysis
Source: Front Med (Lausanne). 2026 May 13;13:1830155. doi: 10.3389/fmed.2026.1830155 (PMC13213865; doi:10.3389/fmed.2026.1830155)
Supplement: Supplementary file 5 [file Table_2.DOCX]

**Supplementary Table 2.** Key Confounding Variables Considered by Selected Studies.

| **Study**  **(year)** | **Brian G.A. Dalton et al. 2017** | **Doan Thuy Nguyen et al. 2024** | **Elke Van Daele et al. 2022** | **Ioannis Karampinis et al. 2017** | **Kazuhiro Noma et al. 2018** | **Masaki Ohi et al. 2017** | **Rao-Jun Luo et al. 2021** | **Xuan-Tong Song et al. 2020** |
| --- | --- | --- | --- | --- | --- | --- | --- | --- |
| **Study type** | RCS | RCS | RCS | RCS | RCS | RCS | RCS | RCS |
| **Baseline characteristics** |  |  |  |  |  |  |  |  |
| Age | Yes | Yes | Yes | Yes | Yes |  | Yes | Yes |
| Gender | Yes | Yes | Yes | Yes | Yes | Yes | Yes | Yes |
| Smoking |  | Yes | Yes | Yes |  |  | Yes |  |
| Comorbidity | Yes | Yes | Yes |  | Yes |  | Yes |  |
| BMI | Yes | Yes | Yes | Yes | Yes |  | Yes |  |
| Preoperative Serum Albumin Levels | Yes |  |  | Yes |  |  |  |  |
| Tumor Location | Yes | Yes | Yes | Yes | Yes | Yes | Yes | Yes |
| T Stage | Yes | Yes | Yes | Yes | Yes | Yes | Yes | Yes |
| Histological Type |  | Yes | Yes |  | Yes |  | Yes |  |
| **Preoperative treatment factors** |  |  |  |  |  |  |  |  |
| NT | Yes | Yes | Yes |  | Yes |  |  |  |
| NACT |  |  | Yes | Yes | Yes |  | Yes |  |
| NCRT |  |  | Yes | Yes | Yes |  | Yes |  |
| **Key Characteristics of the Surgery** |  |  |  |  |  |  |  |  |
| Surgical Approach | Yes | Yes | Yes | Yes | Yes | Yes | Yes | Yes |
| Operating Time | Yes | Yes |  |  | Yes |  | Yes | Yes |
| Intraoperative Blood Loss | Yes |  |  |  |  |  | Yes | Yes |
| Dose of ICG | Yes | Yes |  | Yes | Yes | Yes |  | Yes |

RCS, retrospective cohort study; BMI, body mass index; NT, neoadjuvant therapy; NACT, neoadjuvant chemotherapy; NCRT, neoadjuvant chemoradiotherapy; ICG, indocyanine green.
