# Supplementary material for: The effectiveness of intraoperative indocyanine green fluorescence imaging in preventing anastomotic leakage after minimally invasive esophagectomy for esophageal cancer: a systematic review and meta-analysis
Source: Front Med (Lausanne). 2026 May 13;13:1830155. doi: 10.3389/fmed.2026.1830155 (PMC13213865; doi:10.3389/fmed.2026.1830155)
Supplement: Supplementary file 6 [file Table_3.DOCX]

**Supplementary Table 3.** Judgment of risk of bias by domain for selected studies.

| **Study**  **(year)** |  | **Risk of Bias by Domain** | | | | | | |  | **Overall Risk of Bias** |
| --- | --- | --- | --- | --- | --- | --- | --- | --- | --- | --- |
|  |  | **Confounding** | **Selection of Participants** | **Classification of Interventions** | **Deviations from**  **Intended Interventions** | **Missing**  **Data** | **Measurement**  **Outcomes** | **Selective**  **Reporting of**  **Results** |  |  |
| Brian G.A. Dalton et al. 2017 |  | Moderate risk | Low risk | Low risk | Low risk | Low risk | Low risk | Low risk |  | Moderate risk |
| Doan Thuy Nguyen et al. 2024 |  | Low risk | Low risk | Low risk | Low risk | Low risk | Low risk | Low risk |  | Low risk |
| Elke Van Daele et al. 2022 |  | Low risk | Low risk | Low risk | Low risk | Low risk | Low risk | Low risk |  | Low risk |
| Ioannis Karampinis et al. 2017 |  | Low risk | Low risk | Low risk | Low risk | Low risk | Low risk | Low risk |  | Low risk |
| Kazuhiro Noma et al. 2018 |  | Low risk | Low risk | Low risk | Low risk | Low risk | Low risk | Low risk |  | Low risk |
| Masaki Ohi et al. 2017 |  | Moderate risk | Low risk | Low risk | Low risk | Low risk | Low risk | Low risk |  | Moderate risk |
| Rao-Jun Luo et al. 2021 |  | Moderate risk | Low risk | Low risk | Low risk | Low risk | Low risk | Low risk |  | Moderate risk |
| Xuan-Tong Song et al. 2020 |  | Low risk | Low risk | Low risk | Low risk | Low risk | Low risk | Low risk |  | Low risk |

**Note 1.** Risk of bias was assessed using the Risk Of Bias In Non-randomised Studies - of Interventions (ROBINS-I) tool, with a target trial emulated as: patients with esophageal cancer undergoing minimally invasive esophagectomy were randomly assigned to intraoperative indocyanine green (ICG) fluorescence imaging group or conventional surgery without ICG group, to compare the incidence of postoperative anastomotic leakage.

**Note 2.** A priori core confounding factors for the Confounding domain were defined as: age, gender, smoking, comorbidity, body mass index (BMI), preoperative serum albumin levels, tumor location, T stage, histological type, Preoperative treatment factors, neoadjuvant therapy, neoadjuvant chemotherapy, neoadjuvant chemoradiotherapy, surgical approach, operating time, intraoperative blood loss, dose of ICG.

**Note 3.** For the Measurement of Outcomes domain, all studies were rated as Low risk, as the primary outcome (anastomotic leakage) was defined by objective, internationally recognized clinical, endoscopic or radiological diagnostic criteria, with no subjective bias introduced even without blinded assessment, in accordance with the ROBINS-I official guidance.

**Note 4.** Overall risk of bias rating criteria (Cochrane ROBINS-I standard):

- Low risk: All domains were rated as Low risk;

- Moderate risk: No domains rated as Serious/Critical risk, with one or more domains rated as Moderate risk;

- Serious risk: At least one domain rated as Serious risk, no Critical risk domains;

- Critical risk: At least one domain rated as Critical risk.

**Note 5.** None of the included studies had Serious or Critical risk of bias, and all were eligible for inclusion in the meta-analysis.
