# Supplementary material for: The effectiveness of intraoperative indocyanine green fluorescence imaging in preventing anastomotic leakage after minimally invasive esophagectomy for esophageal cancer: a systematic review and meta-analysis
Source: Front Med (Lausanne). 2026 May 13;13:1830155. doi: 10.3389/fmed.2026.1830155 (PMC13213865; doi:10.3389/fmed.2026.1830155)
Supplement: Supplementary file 7 [file Table_4.DOCX]

**Supplementary Table 4.** Detailed quality assessment of included studies.

| **Included Studies** | **Items of NOS** | | | | | | | | |
| --- | --- | --- | --- | --- | --- | --- | --- | --- | --- |
|  | **Selection** | | | | **Comparability** | **Exposure** | | | **Total** |
|  | Adequacy of the definition of the case | Representativeness of cases | Selection of the control | Definition of the control | Comparability of between cases and controls on basis of the design or analysis | Ascertainment of exposure | Whether exposures to cases and controls were determined using the same method | Nonresponse rate |  |
| Brian G.A. Dalton et al. 2017 |  | **★** | **★** | **★** | **★** |  | **★** | **★** | 6 |
| Doan Thuy Nguyen et al. 2024 | **★** | **★** | **★** | **★** | **★★** | **★** |  |  | 7 |
| Elke Van Daele et al. 2022 | **★** | **★** | **★** | **★** | **★★** | **★** |  |  | 7 |
| Ioannis Karampinis et al. 2017 | **★** | **★** | **★** | **★** | **★★** | **★** |  |  | 7 |
| Kazuhiro Noma et al. 2018 | **★** | **★** | **★** | **★** | **★★** |  | **★** | **★** | 8 |
| Masaki Ohi et al. 2017 | **★** | **★** |  | **★** | **★** | **★** |  | **★** | 6 |
| Rao-Jun Luo et al. 2021 | **★** | **★** | **★** | **★** | **★** | **★** |  |  | 6 |
| Xuan-Tong Song et al. 2020 |  | **★** | **★** | **★** | **★** |  | **★** | **★** | 6 |

A study can be awarded a maximum of one star for each numbered item within the Selection and Exposure categories. A maximum of two stars can be given for Comparability. Study rates ≥6 is eligible for further analysis. NOS, Newcastle-Ottawa Scale.
